# Treatment of Moderate Functional Mitral Regurgitation during Aortic Valve Replacement: A Cohort Study

**DOI:** 10.31083/j.rcm2401005

**Published:** 2023-01-03

**Authors:** Xieraili Tiemuerniyazi, Fei Xu, Yangwu Song, Yifeng Nan, Ziang Yang, Liangcai Chen, Dong Zhao, Wei Zhao, Wei Feng

**Affiliations:** ^1^Department of Cardiovascular Surgery, Fuwai Hospital, National Center for Cardiovascular Diseases, National Clinical Research Center for Cardiovascular Diseases, Chinese Academy of Medical Sciences and Peking Union Medical College, 10037 Beijing, China; ^2^Department of Cardiovascular Surgery, Yunnan Fuwai Cardiovascular Hospital, 650000 Kunming, Yunnan, China

**Keywords:** aortic valve replacement, moderate functional mitral regurgitation, severe aortic valve disease, mitral valve repair, mitral valve replacement

## Abstract

**Background:**

Treatment of moderate functional mitral regurgitation (FMR) during aortic valve replacement (AVR) is controversial. This study aimed to evaluate the effect of different surgical strategies in patients with moderate FMR undergoing AVR.

**Methods:**

A total of 468 patients with moderate FMR undergoing AVR from January 2010 to December 2019 were retrospectively studied comparing 3 different surgical strategies, namely isolated AVR, AVR + mitral valve repair (MVr) and AVR + mitral valve replacement (MVR). Survival was estimated using the Kaplan-Meier method and compared with the log-rank test, followed by inverse probability treatment weighting (IPTW) analysis to adjust the between-group imbalances. The primary outcome was overall mortality.

**Results:**

Patients underwent isolated AVR (35.3%), AVR + MVr (30.3%), or AVR + MVR (34.4%). The median follow-up was 27.1 months. AVR + MVR was associated with better improvement of FMR during the early and follow-up period compared to isolated AVR and AVR + MVr (*p *< 0.001). Compared to isolated AVR, AVR + MVR increased the risk of mid-term mortality (hazard ratio [HR]: 2.13, 95% confidence interval [CI]: 1.01–4.48, *p* = 0.046), which was sustained in the IPTW analysis (HR: 4.15, 95% CI: 1.69–10.15, *p* = 0.002). In contrast, AVR + MVr showed only a tendency to increase the risk of follow-up mortality (HR: 1.63, 95% CI: 0.72–3.67, *p* = 0.239), which was more apparent in the IPTW analysis (HR: 2.54, 95% CI: 0.98–6.56, *p *= 0.054).

**Conclusions:**

In patients with severe aortic valve disease and moderate FMR, isolated AVR might be more reasonable than AVR + MVr or AVR + MVR.

## 1. Introduction 

Functional mitral regurgitation (FMR) is characterized by insufficiency of the mitral valve resulting from left ventricle dysfunction in the absence of primary mitral valve pathology [1]. FMR is not uncommon in patients requiring cardiac surgery. Studies report various degrees of FMR are present in up to 75% of patients undergoing aortic valve replacement (AVR), of which 25% can be associated with moderate FMR [2].

Clinical guidelines recommend mitral valve surgery in patients with severe FMR during AVR [3], while controversies exist on the treatment of moderate FMR. Studies report that moderate FMR might improve after isolated AVR [4], while others suggest that it might not always improve, indicating the necessity for concomitant mitral valve surgery [2, 5]. Results from systematic reviews have reported that moderate FMR tends to improve after isolated AVR [6, 7], but the studies included are of poor methodological quality, and most include moderate FMR patients undergoing isolated AVR. A limited number of studies suggest that double valve replacement might be more hazardous in moderate-to-severe FMR patients [8]. Therefore, the impact of mitral valve surgery during AVR in patients with moderate FMR is unknown.

The aim of this study was to evaluate the effect of different surgical techniques on the prognosis of moderate FMR patients undergoing AVR.

## 2. Materials and Methods

### 2.1 Study Design

In this cohort study, 468 eligible patients hospitalized from January 2010 to December 2019 at Fuwai Hospital (Beijing, China) were retrospectively studied. Three different surgical strategies, isolated AVR, AVR + mitral valve repair (MVr) and AVR + mitral valve replacement (MVR), were compared. This study was conducted in accordance with the Declaration of Helsinki. The Institutional Review Board at our Fuwai Hospital approved the use of clinical data for this study (NO.: 2021-1585) and waived individual informed consents.

### 2.2 Study Population, Definitions and Follow-Up

Patients who were >18 years of age, undergoing AVR with moderate FMR were included. We excluded patients with rheumatic heart disease, infective endocarditis, mitral valve prolapse or with primary lesions on the mitral valve. The decision on whether to perform concomitant mitral valve surgery was made according to the individual surgeon’s judgement through comprehensive evaluation of the patient’s condition.For those with larger left ventricle or mitral annulus, eccentric mitral regurgitation and/or longstanding course of aortic valve disease, surgeons might prefer concomitant mitral valve surgery. All of the patients received median thoracotomy, and underwent the surgery under cardiopulmonary bypass.

The primary outcome was the overall mortality. The secondary outcomes were the major adverse cardiovascular and cerebrovascular events (MACCE), perioperative complications and the changes in echocardiographic characteristics, including the grade of FMR, ejection fraction (EF), left ventricular end-diastolic diameter (LVEDD), and left atrial diameter (LAD).

Moderate FMR was diagnosed using transthoracic echocardiography at least for twice after admission to the hospital and before the surgery. The degree of mitral regurgitation was determined according to the vena contracta and the regurgitant jet area, and were stratified into five entities (0+ = no, 1+ = trivial, 2+ = mild, 3+ = moderate, 4+ = severe). Only patients with moderate FMR were included. All patients underwent transesophageal echocardiography in the operating room before the surgical procedure for the further evaluation of the regurgitant level. However, since the regurgitant level might be underestimated during general anesthesia, transesophageal echocardiography was only used as a reference. Operative death was defined as death within 30 days postoperatively. MACCE was defined as the composite of all-cause death, myocardial infarction, ischemic or hemorrhagic stroke, hospitalization for heart failure and repeat valvular surgery.

Baseline and perioperative characteristics of the patients were obtained from electronic hospital records. Patients were required to return back to the institute for routine re-examination at 3, 6 and 12 months postoperatively. For patients who survived for more than a year, the follow-up was then made annually. Phone call interviews were used for patients who were unavailable for re-examination at our institute.

### 2.3 Statistical Analysis

Continuous variables were presented as mean ± standard deviation (SD) if they follow normal distribution, and tested by one-way analysis of variance (ANOVA). Otherwise, they are presented using medians with the 25th and 75th percentiles and tested by Kruskal-Wallis H test. Categorical variables were presented as numbers (%) and tested by Chi-square test or Fisher exact test, as appropriate. Cumulative survivals were calculated using the Kaplan-Meier method and compared using the log-rank test. Inverse probability treatment weighting (IPTW) analysis was performed to balance the baseline confounders. Variables balanced in the IPTW analysis included age, sex, body mass index, body surface area, preoperative atrial fibrillation, New York Heart Association (NYHA) class III or IV, hypertension, dyslipidemia, coronary artery disease, diabetes mellitus, renal failure, preoperative EF, LVEDD, LAD, severity of tricuspid valve regurgitation, etiology of FMR, type of aortic valve disease, type of aortic prosthesis, and concomitant coronary artery bypass grafting, tricuspid valve surgery, other procedures, and postoperative administration of angiotensin converting enzyme inhibitors/angiotensin-receptor blockers. A standardized mean difference (SMD) <0.2 or *p*-value > 0.05 was considered to indicate adequate balance for between-group differences. The IPTW analysis was achieved using “ipw” R package. A *p* value < 0.05 was considered statistically significant, and Bonferroni correction was applied in the multiple comparisons, as appropriate. Statistical analyses were performed using R 4.1.2 (R Core Team, Vienna, Austria).

## 3. Results

### 3.1 Patient Characteristics and Operative Details

A total of 468 patients undergoing AVR (35.3%), AVR + MVr (30.3%) or AVR + MVR (34.4%) were included. The most commonly used MVr technique was a ring annuloplasty (86.6%), followed by band repair (9.9%) and leaflet repair (3.5%). The mean age was 57.3 ± 12.5 years, and 335 (71.6%) were male. Body mass index, body surface area, and the history of hypertension, preoperative LAD and LVEDD differed among the three groups (*p *< 0.05). The incidence of aortic insufficiency was much lower in the AVR group (*p* = 0.016) (Table 1).

**Table 1.** **Baseline characteristics**.

Variables		AVR (n = 165)	AVR + MVr (n = 142)	AVR + MVR (n = 161)	*p*-value
Age (years), mean ± SD		59.2 ± 12.3	56.4 ± 12.7	56.2 ± 12.4	0.054
Female sex, no (%)		57 (34.5)	32 (22.5)	44 (27.3)	0.062
Body mass index (${\mathrm{kg/m}}^{2}$), median [Q1, Q3]		22.8 [20.1, 25.8]	23.7 ${\mathrm{[21.5, 26.6]}}^{†}$	22.8 ${\mathrm{[20.8, 25.0]}}^{‡}$	0.025
Body surface area ($m^{2}$), median [Q1, Q3]		1.8 [1.6, 1.9]	1.8 [1.7, 2.0]	1.7 [1.6, 1.9]	0.036
Atrial fibrillation, no (%)		17 (10.3)	21 (14.8)	32 (19.9)	0.053
NYHA class III or IV, no (%)		72 (43.6)	80 (56.3)	84 (52.2)	0.073
Hypertension, no (%)		64 (38.8)	66 (46.5)	46 ${\mathrm{(28.6)}}^{‡}$	0.005
Dyslipidemia, no (%)		50 (30.3)	44 (31.0)	33 (20.5)	0.064
Coronary artery disease, no (%)		28 (17.0)	29 (20.4)	20 (12.4)	0.168
Diabetes mellitus, no (%)		22 (13.3)	10 (7.0)	10 ${\mathrm{(6.2)}}^{†}$	0.050
Renal failure, no (%)		4 (2.4)	9 (6.3)	6 (3.7)	0.215
EF (%), median [Q1, Q3]		55.0 [46.0, 60.0]	56.5 [50.0, 62.0]	58.0 [52.0, 61.0]	0.144
LVEDD (mm), median [Q1, Q3]		61.0 [54.0, 71.0]	66.0 ${\mathrm{[61.0, 71.0]}}^{†}$	64.0 ${\mathrm{[59.0, 71.0]}}^{†}$	0.001
LAD (mm), median [Q1, Q3]		42.0 [38.0, 47.0]	45.0 ${\mathrm{[41.0, 51.0]}}^{†}$	46.0 ${\mathrm{[43.0, 50.0]}}^{†}$	<0.001
Aortic valve, no (%)					0.016
	Insufficiency	95 (57.6)	103 ${\mathrm{(72.5)}}^{†}$	110 ${\mathrm{(68.3)}}^{†}$	
	Stenosis	70 (42.4)	39 (27.5)	51 (36.7)	
Tricuspid regurgitation, no (%)					0.358
	No	68 (41.2)	46 (32.4)	57 (35.4)	
	Trivial	30 (18.2)	23 (16.2)	27 (16.8)	
	Mild	53 (32.1)	46 (32.4)	53 (32.9)	
	Moderate	12 (7.3)	25 (17.6)	21 (13.0)	
	Severe	2 (1.2)	2 (1.4)	3 (1.9)	
Etiology of FMR, no ${\mathrm{(\%)}}^{a}$					0.168
	Non-ischemic	28 (17.0)	29 (20.4)	20 (12.4)	
	Ischemic and non-ischemic	137 (83.0)	113 (79.6)	141 (87.6)	

^a^Non-ischemic, severe aortic valve disease with FMR, without history or preoperative angiographic findings of coronary artery disease or; ischemic and non-ischemic, severe aortic valve disease with FMR, with a history of coronary artery disease or >50% stenosis of coronary artery in the preoperative angiographic tests, followed by ventricular regional wall motion abnormality or papillary muscle dysfunction. ^†^*p *< 0.05 vs. AVR after Bonferroni correction; ^‡^*p *< 0.05 vs. AVR + MVr after Bonferroni correction. AVR, aortic valve replacement; LAD, left atrial diameter; EF, ejection fraction; LVEDD, left ventricular end-diastolic diameter; MVr, mitral valve repair; MVR, mitral valve replacement; NYHA, New York Heart Association; SD, standard deviation.

Less patients in the AVR group received tricuspid valve repair (which included DeVaga’s annuloplasty, Ring annuloplasty and Kay’s annuloplasty). Both AVR + MVr and AVR + MVR increased the duration of cardiopulmonary bypass and the cross-clamp time (Table 2).

**Table 2.** **Operative characteristics**.

Variables		AVR (n = 165)	AVR + MVr (n = 142)	AVR + MVR (n = 161)	*p*-value
Prosthetic valve type, no (%)					0.278
Mechanical		106 (64.2)	100 (70.4)	116 (72.1)	
Bioprosthetic		59 (35.8)	42 (29.6)	45 (28.0)	
Coronary artery bypass grafting, no (%)		27 (16.4)	23 (16.2)	16 (9.9)	0.172
Tricuspid valve repair, no (%)		6 (3.6)	34 ${\mathrm{(23.9)}}^{†}$	64 ${\mathrm{(39.8)}}^{†,‡}$	<0.001
	DeVaga’s annuloplasty	3	19	23	
	Ring annuloplasty	2	6	33	
	Kay’s annuloplasty	1	9	8	
Other ${\mathrm{procedures}}^{§}$		13 (7.9)	14 (9.9)	8 (4.97)	0.264
Cardiopulmonary bypass (min), median [Q1, Q3]		98.0 [78.0, 131.0]	141.0 ${\mathrm{[122.0, 183.0]}}^{†}$	146.0 ${\mathrm{[121.0, 182.0]}}^{†}$	<0.001
Cross-clamp time (min), median [Q1, Q3]		71.0 [56.0, 99.5]	110.0 ${\mathrm{[92.0, 136.0]}}^{†}$	111.0 ${\mathrm{[90.0, 143.0]}}^{†}$	<0.001

^†^*p *< 0.05 vs. AVR after Bonferroni correction; ^‡^*p *< 0.05 vs. AVR + MVr after Bonferroni correction; ^§^Included repair of atrial septal defect or ventricular septal defect, removal of left atrial thrombus, etc. AVR, aortic valve replacement; MVr, mitral valve repair; MVR, mitral valve replacement.

### 3.2 Early Postoperative Outcomes

Nine of the patients had an operative death. AVR + MVR increased the risk of operative death (*p *< 0.001) and reoperation for bleeding (*p <* 0.001) (Table 3).

**Table 3.** **Early postoperative characteristics**.

Variables		AVR (n = 165)	AVR + MVr (n = 142)	AVR + MVR (n = 161)	*p*-value
Usage of ACEI/ARB, no (%)		21 (12.7)	22 (15.5)	15 (9.3)	0.262
Operative death, no (%)		0	1 (0.7)	8 ${\mathrm{(5.0)}}^{†,‡}$	0.001
Reoperation for bleeding, no (%)		0	1 (0.7)	10 ${\mathrm{(6.2)}}^{†,‡}$	<0.001
New-onset stroke, no (%)		0	0	2 (1.2)	0.208
New-onset AF, no (%)		7 (4.2)	8 (5.6)	13 (8.1)	0.338
Acute kidney injury, no (%)		14 (8.5)	10 (7.0)	13 (8.1)	0.338
ΔEF (%), median [Q1, Q3]		–3.0 [–8.0, 5.0]	–4.0 ${\mathrm{[–9.0, 1.0]}}^{†}$	–5.0 ${\mathrm{[–12.0, 1.0]}}^{†}$	0.002
ΔLVEDD (mm), median [Q1, Q3]		–9.0 [–13.0, –5.0]	–12.0 ${\mathrm{[–16.0, –6.0]}}^{†}$	–11.0 ${\mathrm{[–15.0, –6.0]}}^{†}$	0.009
ΔLAD (mm), median [Q1, Q3]		–7.0 [–10.0, –3.0]	–7.0 [–12.0, –3.0]	–5.0 [–10.0, –2.0]	0.057
Tricuspid regurgitation, no (%)					0.626
	No	90 (54.5)	88 (62.0)	99 (61.5)	
	Trivial/Mild	72 (43.6)	51 (35.9)	60 (37.3)	
	Moderate	3 (1.8)	3 (2.1)	2 (1.2)	
FMR, no (%)					<0.001
	No	101 (61.2)	88 (62.0)	157 (97.5)	
	Trivial/mild	60 (36.4)	51 (35.9)	4 (2.5)	
	Moderate	4 (2.4)	3 (2.1)	0	

^†^*p *< 0.05 vs. AVR after Bonferroni correction; ^‡^*p *< 0.05 vs. AVR + MVr after Bonferroni correction; Δ Change compared to the baseline. ACEI/ARB, angiotensin converting enzyme inhibitors/angiotensin-receptor blockers; AVR, aortic valve replacement; LAD, left atrial diameter; EF, ejection fraction; LVEDD, left ventricular end-diastolic diameter; MVr, mitral valve repair; MVR, mitral valve replacement; SD, standard deviation.

Postoperative echocardiograms were performed prior to discharge. Compared to baseline, isolated AVR had less decrease in LVEDD (*p* = 0.009) and EF (*p* = 0.002) than the other two groups, while more significant decrease in FMR degree was observed in the AVR + MVR group (*p *< 0.001). However, there was no significant difference in LAD (*p* = 0.057) among the three groups (Table 3).

### 3.3 Follow-Up Outcomes

**Fig. 1.**
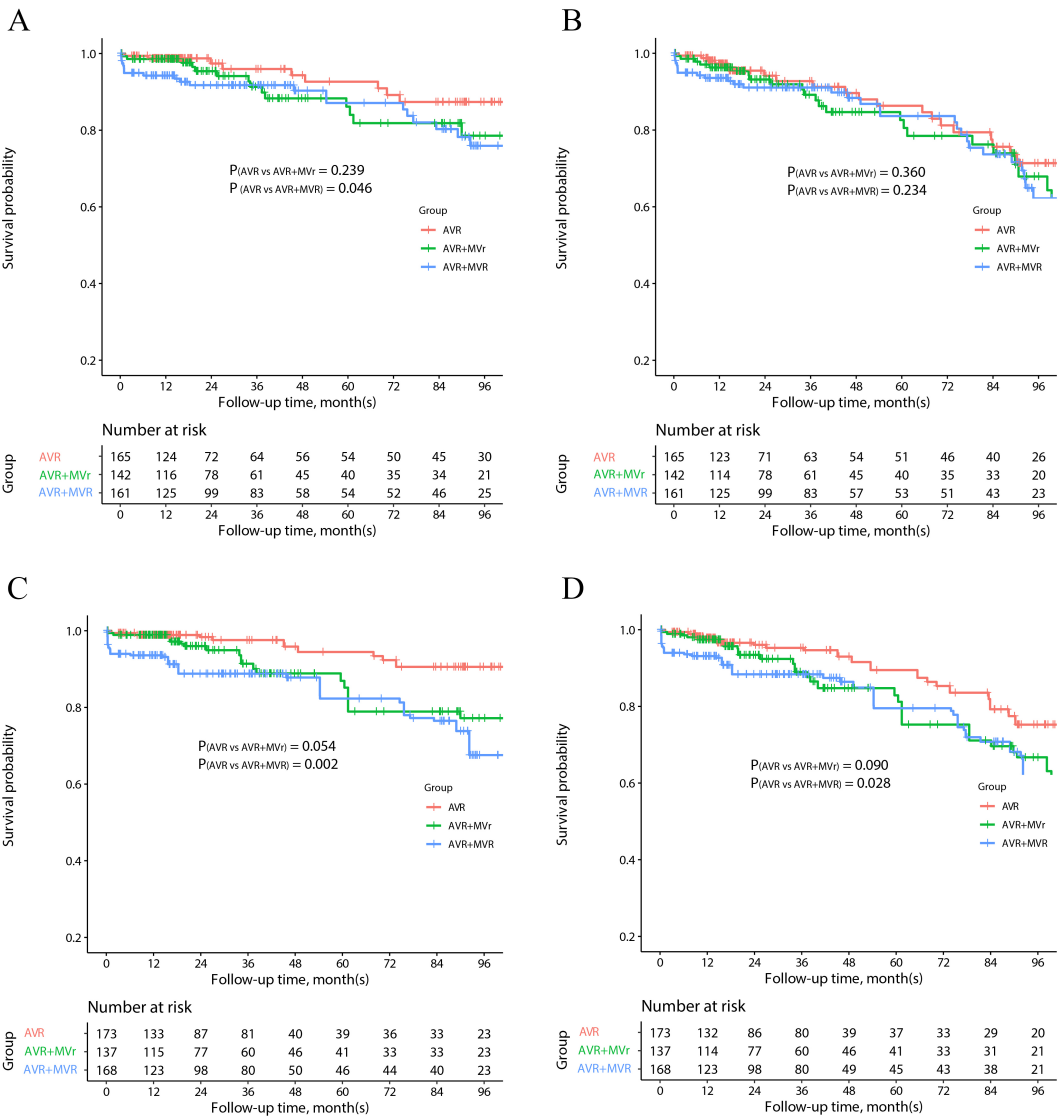
**Survival outcomes of overall cohort**. Kaplan-Meier estimates of overall and MACCE-free survival in the unmatched (A,B) and IPTW analysis (C,D). AVR, aortic valve replacement; IPTW, inverse probability treatment weighting; MACCE, major adverse cardiovascular and cerebrovascular events; MVr, mitral valve repair; MVR, mitral valve replacement.

The median follow-up was 27.1 [13.0, 85.5] months. During follow-up, 47 of the patients died, and the most common cause was cardiac death, while MACCE was observed in 77 patients (Table 4). AVR + MVR increased the risk of follow-up mortality (hazard ratio [HR]: 2.13, 95% confidence interval [CI]: 1.01–4.48, *p* = 0.046), while AVR + MVr showed similar survival (HR: 1.63, 95% CI: 0.72–3.67, *p* = 0.239) with isolated AVR. Both AVR + MVr (HR: 1.32, 95% CI: 0.73–2.36, *p* = 0.360) and AVR + MVR (HR: 1.40, 95% CI: 0.81–2.43, *p* = 0.234) did not increase the risk of MACCE (Fig. 1).

**Table 4.** **Follow-up outcomes**.

Variables		AVR (n = 165)	AVR + MVr (n = 142)	AVR + MVR (n = 161)	*p*-value $1^{a}$	*p*-value $2^{b}$
Death, no (%)		10 (6.1)	14 (9.9)	23 (14.3)	0.239	0.046
	Cardiac	8 (4.9)	13 (9.2)	17 (10.6)		
	Stroke	1 (0.6)	0	3 (1.9)		
	Other causes	1 (0.6)	1 (0.7)	3 (1.9)		
MACCE, no (%)		21 (12.7)	24 (16.9)	32 (19.9)	0.360	0.234
	All-cause death	10 (6.1)	11 (7.8)	18 (11.2)		
	Myocardial infarction	1 (0.6)	1 (0.7)	1 (0.6)		
	Stroke	5 (3.0)	6 (4.2)	2 (1.2)		
	Repeat surgery	0	1 (0.7)	4 (2.5)		
	Hospitalization for heart failure	5 (3.0)	5 (3.5)	7 (4.4)		

^a^*p*-value of log-rank test for AVR vs. AVR + MVr; ^b^*p*-value of log-rank test for AVR vs. AVR + MVR. AVR, aortic valve replacement; MACCE, major adverse cardiovascular and cerebrovascular events; MVr, mitral valve repair; MVR, mitral valve repair.

Follow-up echocardiographic results from 3 to 12 months after surgery were obtained for 72.4% of the patients. The median follow-up time for echocardiography was 3.7 [3.2, 6.8] months. AVR + MVR showed the least improvement in EF (*p* = 0.006), but had significantly better improvement in the degree of FMR (*p *< 0.001) than the patients in the other two groups (Table 5).

**Table 5.** **Follow-up echocardiographic results**.

Variables		AVR (n = 119)	AVR + MVr (n = 98)	AVR + MVR (n = 121)	*p*-value
ΔEF (%), median [Q1, Q3]		3.0 [–2.0, 10.0]	4.0 [–1.0, 10.0]	0 ${\mathrm{[–5.0, 7.0]}}^{†,‡}$	0.006
ΔLVEDD (mm), median [Q1, Q3]		–12.0 [–17.0, –8.0]	–15.0 [–2.0, –10.0]	–13.0 [–19.0, –7.0]	0.055
ΔLAD (mm), median [Q1, Q3]		–5.0 [–9.0, –2.0]	–7.0 [–11.0, –2.0]	–5.0 [–10.0, 0]	0.213
Tricuspid regurgitation, no (%)					0.993
	No	64 (53.8)	52 (53.1)	62 (51.2)	
	Trivial/Mild	48 (40.3)	41 (41.8)	52 (43.0)	
	Moderate	7 (5.9)	5 (5.1)	7 (5.8)	
FMR, no (%)					<0.001
	No	78 (65.5)	69 (70.4)	117 (96.7)	
	Trivial/Mild	36 (30.3)	22 (22.4)	2 (1.7)	
	Moderate	5 (4.2)	7 (7.1)	2 (1.7)	

^†^*p *< 0.05 vs. AVR after Bonferroni correction; ^‡^*p *< 0.05 vs. AVR + MVr after Bonferroni correction; Δ Change compared to the baseline. FMR, functional mitral regurgitation; LAD, left atrial diameter; EF, ejection fraction; LVEDD, left ventricular end-diastolic diameter; SD, standard deviation.

### 3.4 IPTW Analysis

In the IPTW analysis, all of the baseline characteristics were considered to be well-balanced among the three groups (**Supplementary Table 1**). Similar to the unmatched cohort, AVR + MVr and AVR + MVR increased the duration of cardiopulmonary bypass and the cross-clamp time, although the difference was not statistically significant.

In the early postoperative results, significant differences were observed in the operative death among the three groups (*p* = 0.007), as well as the rate of reoperation for bleeding (*p* = 0.002). In the multiple comparisons, AVR + MVR was observed to be associated with increased operative death and reoperation for bleeding compared to the isolated AVR and AVR + MVr groups (*p *< 0.05 for all after Bonferroni correction). AVR + MVR resulted in less reduction in postoperative LAD size (*p *< 0.001), but was associated with better improvement of FMR (*p *< 0.001) (**Supplementary Table 1**).

On long-term follow-up, AVR + MVR was associated with increased mortality (HR: 4.15, 95% CI: 1.69–10.15, *p* = 0.002) and increased risk of MACCE (HR: 2.20, 95% CI: 1.09–4.42, *p* = 0.028) when compared to isolated AVR (Fig. 1). AVR + MVr showed a tendency to increase the risk of follow-up mortality (HR: 2.54, 95% CI: 0.98–6.56, *p* = 0.054) and MACCE (HR: 1.83, 95% CI: 0.91–3.69, *p* = 0.090) compared to isolated AVR, although it did not reach statistical significance. On follow-up echocardiograms, AVR + MVR showed less reduction in the size of LAD (*p *< 0.001), but better improvement of FMR (*p *< 0.001) (**Supplementary Table 2**).

### 3.5 Subgroup Analysis

Patients were further stratified into two subgroups according to the type of aortic valve disease, aortic insufficiency and aortic stenosis. Baseline and operative characteristics were balanced through IPTW analysis (**Supplementary Tables 3,4**).

**Fig. 2.**
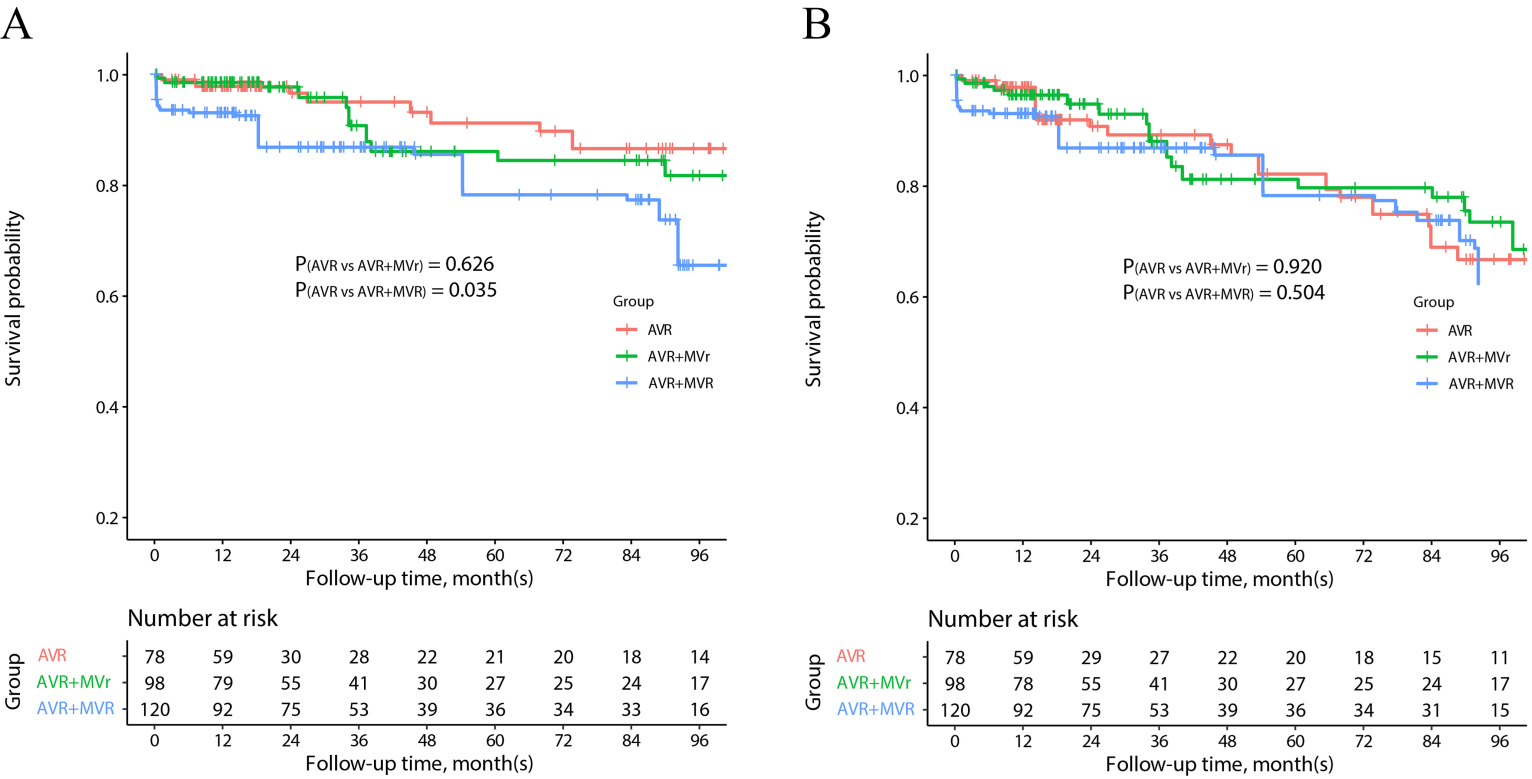
**Survival outcomes of aortic insufficiency patients**. Kaplan-Meier estimates of overall (A) and MACCE-free (B) survival in the IPTW analysis. AVR, aortic valve replacement; IPTW, inverse probability treatment weighting; MACCE, major adverse cardiovascular and cerebrovascular events; MVr, mitral valve repair; MVR, mitral valve replacement.

In the subgroup of aortic insufficiency, early postoperative results were consistent with those of the overall cohort (**Supplementary Table 3**). Both AVR + MVr (*p* = 0.727) and AVR + MVR (*p* = 0.407) did not increase the risk of follow-up MACCE (Fig. 2), while AVR + MVR was observed to be associated with increased follow-up mortality (*p* = 0.035).

**Fig. 3.**
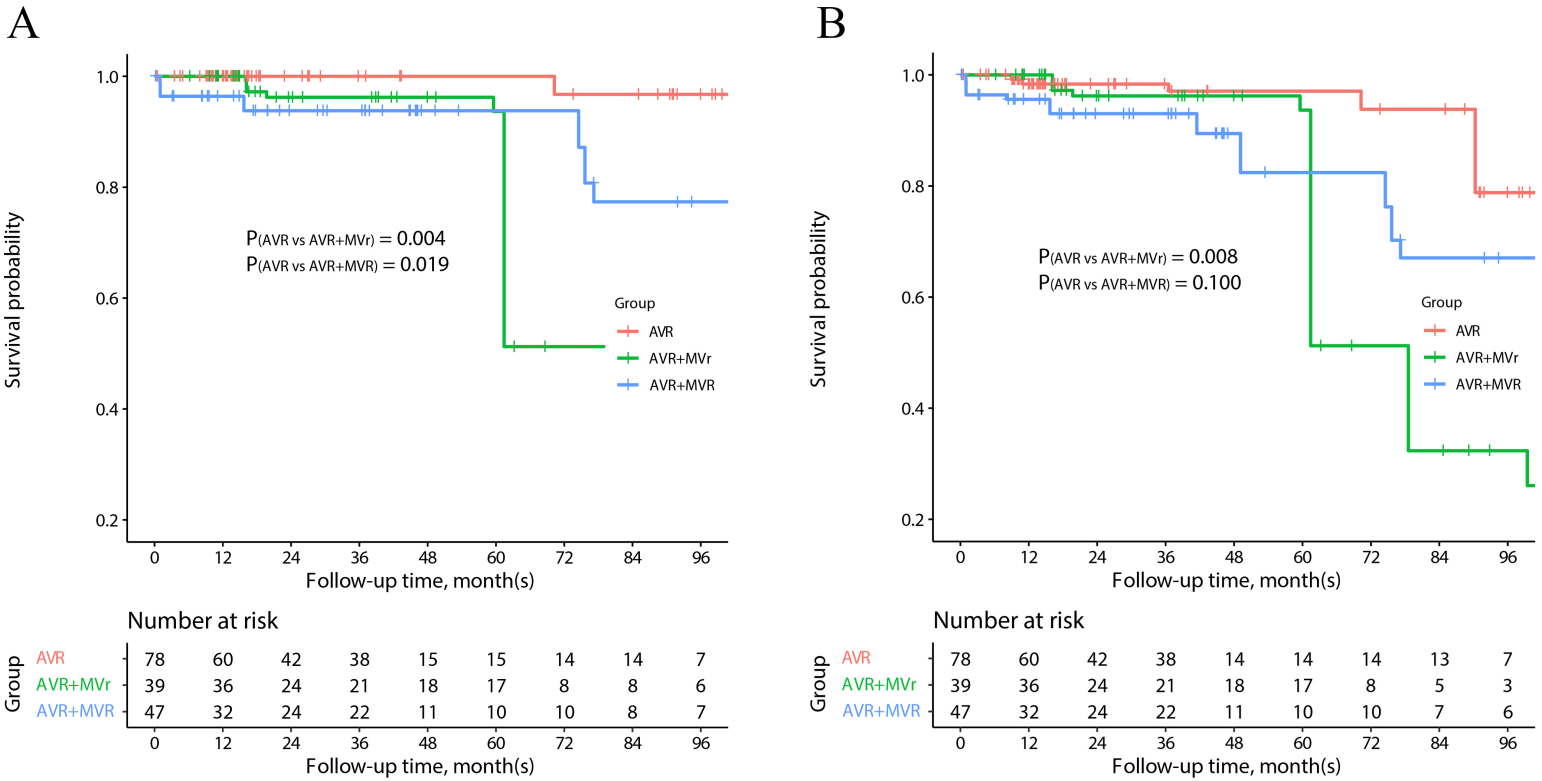
**Survival outcomes of aortic stenosis patients**. Kaplan-Meier estimates of overall (A) and MACCE-free (B) survival in the IPTW analysis. AVR, aortic valve replacement; IPTW, inverse probability treatment weighting; MACCE, major adverse cardiovascular and cerebrovascular events; MVr, mitral valve repair; MVR, mitral valve replacement.

In the aortic stenosis subgroup (**Supplementary Table 4**), AVR + MVR was observed to be associated with an increased risk of postoperative new-onset atrial fibrillation (*p* = 0.004). AVR + MVr also increased the risk of mortality (*p* = 0.004) and MACCE (*p* = 0.006), while AVR + MVR was associated with a higher risk of mortality (*p* = 0.019) but not MACCE (*p* = 0.100) during the follow-up period (Fig. 3).

## 4. Discussion

In this study, we observed that as compared to isolated AVR, AVR + MVR was associated with an increased risk of postoperative and mortality as well as MACCE in patients with severe aortic valve disease complicated by moderate FMR. In contrast, AVR + MVr showed only a trend to increase the risk of follow-up mortality and MACCE. Subgroup analyses revealed similar outcomes.

### 4.1 Controversies for the Treatment of Moderate FMR in Severe Aortic Valve Diseases

Unlike primary mitral valve disease, moderate or less than moderate FMR might improve or disappear after isolated AVR. Previous studies found that improvement of moderate FMR after isolated AVR can be as high as 95% [6, 7]. However, several studies report that moderate FMR, especially residual FMR after isolated AVR [5, 9, 10], may compromise long-term prognosis, indicating the necessity for concomitant mitral valve surgery, while others suggest the opposite results [4, 11, 12]. Therefore, controversies exist regarding whether to operate on the mitral valve in patients with moderate FMR during AVR. In this study, we observed that moderate FMR had improved in the majority of patients immediately after AVR, irregardless of a concomitant mitral valve intervention. This might be due to the pathophysiological mechanism of FMR. In patients with severe aortic valve disease, FMR can be directly caused by the expansion of the mitral annulus, which is attributed to the enlargement and pressure increase of the left ventricle. Correction of the aortic valve abnormalities can result in the reduction of left ventricular size and pressure, resulting in an improvement of moderate FMR after isolated AVR. However, moderate FMR persisted in several patients during both the early and mid-term follow-up.

### 4.2 Impact of Mitral Valve Surgery on Survival of Patients with Moderate FMR Undergoing AVR

Double valve replacement is associated with increased mortality in patients with primary mitral valve disease [8, 13]. Several studies have evaluated the effect of mitral valve surgery in FMR patients. Studies report that in severe ischemic FMR patients, MVR prevents recurrent mitral regurgitation and reduces heart failure events but not mortality compared to MVr [14, 15]. However, few studies have compared the outcomes of different operative techniques in patient with moderate FMR undergoing AVR. In our study, we found that AVR + MVR increased the risk of operative and mid-term mortality in moderate FMR patients. These results are consistent with previous studies on primary mitral valve disease [8, 13]. AVR + MVR also increased the risk of reoperation for bleeding, and had a higher risk of MACCE in the IPTW analysis.

MVr is another surgical option for moderate FMR. However, in a previous study, MVr did not improve survival or adverse events in patients with moderate ischemic FMR [16]. In this study, we observed that there was a non-statistical increase in the incidence of adverse events after AVR + MVr. Therefore, isolated AVR, rather than AVR + MVr or AVR + MVR, might be a more reasonable procedure in some patients with moderate FMR requiring an AVR.

### 4.3 Impact of Aortic Valve Etiology on the Prognosis of Moderate FMR Patients

Most of the prior studies include patients with aortic stenosis and moderate FMR [17, 18, 19]. However, researchers also raise their concerns on the impact of different aortic valve etiology on long-term outcomes [20]. The pathophysiological mechanisms of aortic insufficiency and aortic stenosis in patients with FMR are different. In aortic insufficiency and FMR, dilatation of the left ventricle can be severe and the pattern of hypertrophic remodeling is eccentric [21], which is attributed to increases in preload, and worsening left ventricular performance [22]. In patients with aortic stenosis, the long-standing increases in afterload and left ventricular pressure gradient causes hypertrophic remodeling of the left ventricle [21, 23]. The left ventricle decompensates over time, and results in left ventricular dilation and systolic dysfunction, leading to mitral annular dilatation resulting in FMR [24]. As a consequence, the long-term prognosis may differ in patients with aortic insufficiency compared to aortic stenosis with moderate FMR.

In this study, we stratified patients into two subgroups, aortic insufficiency and aortic stenosis. In the aortic insufficiency subgroup, AVR + MVR was observed to be associated with an increased risk of operative and follow-up mortality, while both AVR + MVr and AVR + MVR increased the risk of follow-up mortality in the aortic stenosis patients. In addition, AVR + MVr also increased the risk of follow-up MACCE. Therefore, isolated AVR might be more reasonable regardless of the etiology of the aortic valve disease.

### 4.4 Study Limitations

This study has several limitations. First, this was a retrospective cohort study from a single center. Therefore, the potential for selection bias resulting from the study design cannot be avoided. Second, the sample size was limited, especially in the subgroup analyses, which might have compromised the statistical power. In addition, even though the IPTW analysis balanced the baseline characteristics of the patients, unmeasured confounders could still be present. Finally, follow-up echocardiographic results were not available for all of the patients who survived during the follow-up, which might have influenced the long-term outcomes of the 3 patient groups.

## 5. Conclusions

In patients with severe aortic valve disease with moderate FMR, isolated AVR might be more reasonable than AVR + MVr or AVR + MVR. Additional studies with larger sample sizes and longer follow-up are needed to resolve this issue.

## Data Availability

The datasets used and/or analyzed during the current study are available from the corresponding author on reasonable request.

## Abbreviations

AVR, aortic valve replacement; FMR, functional mitral regurgitation; IPTW, inverse probability treatment weighting; LAD, left atrial diameter; LVEDD, left ventricular end-diastolic diameter; EF, ejection fraction; MACCE, major adverse cardiovascular and cerebrovascular events; MVr, mitral valve repair; MVR, mitral valve replacement.

## Supplementary Material

Supplementary material associated with this article can be found, in the online version, at https://doi.org/10.31083/j.rcm2401005.
