# Supplementary material for: Treatment of Moderate Functional Mitral Regurgitation during Aortic Valve Replacement: A Cohort Study
Source: Rev Cardiovasc Med. 2023 Jan 3;24(1):5. doi: 10.31083/j.rcm2401005 (PMC11270475; doi:10.31083/j.rcm2401005)
Supplement: Supplementary file 1 [file 2153-8174-24-1-005-s1.zip › 2153-8174-24-1-5-s1.docx]

**Table S1.** Baseline, operative and postoperative characteristics in the IPTW analysis.

| Variables | AVR (n= 173.07) | AVR+MVr (n = 137.31) | AVR+MVR (n= 167.62) | P-value | SMD |
| --- | --- | --- | --- | --- | --- |
| ***Preoperative*** | | | | | |
| Age (years), mean ± SD | 56.9 ± 11.3 | 57.2 ± 12.4 | 58.3 ± 11.9 | 0.648 | 0.079 |
| Female sex, no (%) | 73.5 (42.5) | 39.4 (28.7) | 45.2 (26.9) | 0.262 | 0.220 |
| Body mass index (kg/m^2^), median [Q1, Q3] | 22.5 [21.5, 25.5] | 23.2 [20.8, 26.3] | 22.9 [21.1, 25.2] | 0.856 | 0.028 |
| Body surface area (m^2^), median [Q1, Q3] | 1.7 [1.5, 1.89] | 1.8 [1.6, 1.9] | 1.7 [1.7, 1.9] | 0.690 | 0.141 |
| Atrial fibrillation, no (%) | 48.3 (27.9) | 21.1 (15.4) | 27.5 (16.4) | 0.380 | 0.206 |
| NYHA class III or IV, no (%) | 100.9 (58.3) | 73.0 (53.1) | 84.3 (50.3) | 0.634 | 0.108 |
| Hypertension, no (%) | 90.0 (52.0) | 55.0 (40.1) | 76.5 (45.7) | 0.478 | 0.160 |
| Dyslipidemia, no (%) | 42.5 (24.6) | 36.8 (26.8) | 52.8 (31.5) | 0.613 | 0.103 |
| Coronary artery disease, no (%) | 22.3 (12.9) | 21.7 (15.8) | 28.2 (16.8) | 0.707 | 0.074 |
| Diabetes mellitus, no (%) | 13.5 (7.8) | 9.7 (7.1) | 20.4 (12.1) | 0.418 | 0.115 |
| Renal failure, no (%) | 3.9 (2.3) | 6.1 (4.5) | 4.4 (2.6) | 0.521 | 0.081 |
| EF (%), median [Q1, Q3] | 57.6 [50.0, 60.0] | 56.0 [48.0, 61.0] | 56.0 [50.0, 60.0] | 0.448 | 0.101 |
| LVEDD (mm), median [Q1, Q3] | 67.0 [58.0, 71.6] | 65.0 [60.0, 71.0] | 63.0 [59.0, 69.0] | 0.624 | 0.070 |
| LAD (mm), median [Q1, Q3] | 44.4 [40.0, 48.0] | 44.2 [40.0, 50.0] | 45.0 [41.0, 48.0] | 0.811 | 0.075 |
| Aortic valve, no (%) | | | | 0.328 | 0.194 |
| Insufficiency | 89.3 (51.6) | 90.3 (65.8) | 106.8 (63.7) | | |
| Stenosis | 83.8 (48.4) | 47.0 (34.2) | 60.8 (36.3) | | |
| Tricuspid regurgitation, no (%) | | | | 0.866 | 0.170 |
| No | 56.1 (32.4) | 52.0 (37.9) | 61.3 (36.6) | | |
| Trivial | 25.1 (14.5) | 22.9 (16.7) | 26.2 (15.6) | | |
| Mild | 75.7 (43.8) | 44.2 (32.2) | 60.9 (36.3) | | |
| Moderate | 13.6 (7.9) | 16.3 (11.9) | 17.2 (10.3) | | |
| Severe | 2.5 (1.4) | 1.9 (1.4) | 2.0 (1.2) | | |
| Etiology of FMR, no (%) ^a^ | | | | 0.707 | 0.074 |
| Non-ischemic | 150.8 (87.1) | 115.6 (84.2) | 139.4 (83.2) | | |
| Ischemic and non-ischemic | 22.3 (12.9) | 21.7 (15.8) | 28.2 (16.8) | | |
| ***Operative*** | | | | | |
| Mechanical valve, no (%) | 132.4 (76.5) | 93.1 (67.8) | 114.7 (68.5) | 0.408 | 0.130 |
| CABG, no (%) | 19.2 (11.1) | 18.6 (13.5) | 25.3 (15.1) | 0.676 | 0.079 |
| Tricuspid valve repair, no (%) | 43.4 (25.1) | 31.0 (22.5) | 34.2 (20.4) | 0.825 | 0.074 |
| Other procedures, no (%) | 11.0 (6.4) | 11.2 (8.2) | 15.0 (8.9) | 0.765 | 0.064 |
| Cardiopulmonary bypass (min), median [Q1, Q3] | 114.0 [86.6, 204.3] | 136.5 [121.4, 180.0] | 140.3 [119.4, 174.2] | 0.496 | 0.158 |
| Cross-clamp time (min), median [Q1, Q3] | 81.7 [61.6, 150.2] | 105.3 [91.0, 136.0] | 106.0 [86.3, 133.1] | 0.345 | 0.228 |
| ***Early postoperative*** | | | | | |
| Usage of ACEI/ARB, no (%) | 18.0 (10.4) | 15.9 (11.5) | 27.5 (16.4) | 0.434 | 0.118 |
| Operative death, no (%) | 0 | 0.8 (0.6) | 10.0 (5.9) | 0.007 | 0.256 |
| Reoperation for bleeding, no (%) | 0 | 1.1 (0.8) | 12.3 (7.3) | 0.002 | 0.287 |
| New-onset stroke, no (%) | 0 | 0 | 1.1 (0.7) | 0.555 | 0.077 |
| New-onset AF, no (%) | 4.6 (2.7) | 6.8 (4.9) | 16.7 (10.0) | 0.029 | 0.205 |
| Acute kidney injury, no (%) | 13.2 (7.6) | 9.8 (7.2) | 21.9 (13.1) | 0.360 | 0.131 |
| ΔEF (%), median [Q1, Q3] | -5.0 [-12.3, 2.1] | -3.0 [-8.0, 2.0] | -3.2 [-10.3, 3.0] | 0.764 | 0.102 |
| ΔLVEDD (mm), median [Q1, Q3] | -11.0 [-16.2, -7.0] | -11.0 [-15.0, -5.0] | -11.0 [-14.0, -6.0] | 0.923 | 0.064 |
| ΔLAD (mm), median [Q1, Q3] | -7.5 [-11.0, -6.0] | -7.0 [-12.0, -2.5] | -4.0 [-8.0, -0.9] | < 0.001 | 0.408 |
| Tricuspid regurgitation, no (%) | | | | 0.732 | 0.144 |
| No | 105.6 (61.0) | 73.0 (53.2) | 101.7 (60.7) |  |  |
| Trivial/mild | 65.8 (38.0) | 61.5 (44.8) | 62.2 (37.1) |  |  |
| Moderate | 1.7 (1.0) | 2.8 (2.1) | 3.7 (2.2) |  |  |
| Mitral regurgitation, no (%) | | | | < 0.001 | 0.662 |
| No | 122.7 (70.9) | 87.6 (63.8) | 165.3 (98.6) |  |  |
| Trivial/mild | 47.4 (27.4) | 46.9 (34.1) | 2.3 (1.4) |  |  |
| Moderate | 3.0 (1.7) | 2.8 (2.1) | 0 |  |  |

^a^ Non-ischemic: severe aortic valve disease with FMR, without history or preoperative angiographic findings of coronary artery disease or; ischemic and non-ischemic: severe aortic valve disease with FMR, with a history of coronary artery disease or >50% stenosis of coronary artery in the preoperative angiographic tests, followed by ventricular regional wall motion abnormality or papillary muscle dysfunction.

Δ Change compared to the baseline.

ACEI/ARB, angiotensin converting enzyme inhibitors / angiotensin-receptor blockers; AVR, aortic valve replacement; CABG, coronary artery bypass grafting; CPB, cardiopulmonary bypass; EF, ejection fraction; FMR, functional mitral regurgitation; LAD, left atrial diameter; LVEDD, left ventricular end-diastolic diameter; MVr, mitral valve repair; MVR, mitral valve replacement; NYHA, New York Heart Association; SD, standard deviation; SMD, standardized mean difference.

**Table S2.** Follow-up echocardiographic results in the IPTW analysis.

| Variables | AVR (n=137.3) | AVR+MVr (n=98.3) | AVR+MVR (n=118.9) | P-value | SMD |
| --- | --- | --- | --- | --- | --- |
| ΔEF (%), median [Q1, Q3] | -1.0 [-4.6, 5.0] | 5.0 [-1.0, 12.0] | 0.9 [-4.4, 8.0] | 0.025 | 0.285 |
| ΔLVEDD (mm), median [Q1, Q3] | -17.0 [-25.2, -9.0] | -15.0 [-20.0, -10.0] | -13.0 [-18.0, -7.0] | 0.376 | 0.228 |
| ΔLAD (mm), median [Q1, Q3] | -8.0 [-45.2, -4.4] | -6.0 [-10.0, -2.0] | -4.5 [-9.0, 0.0] | 0.027 | 0.575 |
| Tricuspid regurgitation, no (%) | | | | 0.429 | 0.226 |
| No | 87.2 (63.6) | 52.0 (52.9) | 59.4 (49.9) |  |  |
| Trivial/mild | 45.6 (33.3) | 42.0 (42.8) | 50.2 (42.2) |  |  |
| Moderate | 4.4 (3.2) | 4.3 (4.3) | 9.4 (7.9) |  |  |
| FMR, no (%) | | | | < 0.001 | 0.905 |
| No | 66.6 (48.5) | 70.8 (72.1) | 115.9 (97.4) | | |
| Trivial/mild | 67.0 (48.8) | 20.8 (21.2) | 1.5 (1.3) | | |
| Moderate | 3.7 (2.7) | 6.7 (6.8) | 1.5 (1.3) | | |

Δ Change of echocardiographic characteristics compared to the baseline.

AVR, aortic valve replacement; EF, ejection fraction; FMR, functional mitral regurgitation; LAD, left atrial diameter; LVEDD, left ventricular end-diastolic diameter; MVr, mitral valve repair; MVR, mitral valve replacement; SD, standard deviation; SMD, standardized mean difference.

**Table S3.** Baseline, operative and postoperative characteristics of the aortic insufficiency subgroup in the IPTW analysis.

| Variables | AVR  (n = 78.03) | AVR+MVr  (n = 98.45) | AVR+MVR  (n = 119.58) | P-value | SMD |
| --- | --- | --- | --- | --- | --- |
| ***Preoperative*** | | | | | |
| Age (years), mean ± SD | 56.3 ± 12.0 | 55.4 ± 12.8 | 56.6 ± 12.7 | 0.821 | 0.064 |
| Female sex, no (%) | 17.8 (22.8) | 22.9 (23.2) | 26.3 (22.0) | 0.982 | 0.019 |
| Body mass index (kg/m^2^), median [Q1, Q3] | 24.0 [21.1, 27.7] | 23.5 [21.3, 26.5] | 23.9 [21.5, 26.6] | 0.802 | 0.120 |
| Body surface area (m^2^), median [Q1, Q3] | 1.8 [1.7, 1.9] | 1.8 [1.7, 2.0] | 1.8 [1.7, 1.9] | 0.695 | 0.138 |
| Atrial fibrillation, no (%) | 10.1 (12.9) | 17.1 (17.4) | 20.2 (16.9) | 0.722 | 0.084 |
| NYHA class III or IV, no (%) | 35.8 (45.9) | 52.4 (53.2) | 63.3 (53.0) | 0.692 | 0.098 |
| Hypertension, no (%) | 35.8 (45.9) | 44.6 (45.3) | 61.0 (51.0) | 0.771 | 0.076 |
| Dyslipidemia, no (%) | 23.2 (29.8) | 28.1 (28.5) | 39.6 (33.2) | 0.831 | 0.067 |
| Coronary artery disease, no (%) | 11.9 (15.2) | 15.3 (15.6) | 20.3 (17.0) | 0.950 | 0.032 |
| Diabetes mellitus, no (%) | 6.3 (8.1) | 5.4 (5.4) | 12.8 (10.7) | 0.482 | 0.131 |
| Renal failure, no (%) | 0.8 (1.1) | 4.5 (4.6) | 4.0 (3.3) | 0.294 | 0.144 |
| EF (%), median [Q1, Q3] | 59.7 [48.6, 62.0] | 55.3 [50.0, 61.0] | 56.0 [50.0, 60.0] | 0.488 | 0.120 |
| LVEDD (mm), median [Q1, Q3] | 67.1 [61.0, 71.0] | 67.0 [61.0, 73.0] | 66.7 [60.7, 76.0] | 0.919 | 0.062 |
| LAD (mm), median [Q1, Q3] | 46.0 [40.0, 49.0] | 45.0 [41.0, 50.0] | 45.0 [41.0, 48.2] | 0.985 | 0.022 |
| Tricuspid regurgitation, no (%) | | | | 0.967 | 0.170 |
| No | 31.6 (40.5) | 37.7 (38.2) | 52.9 (44.2) | | |
| Trivial | 12.8 (16.5) | 16.8 (17.1) | 17.9 (15.0) | | |
| Mild | 27.9 (35.7) | 31.4 (31.9) | 37.1 (31.0) | | |
| Moderate | 4.4 (5.7) | 11.1 (11.2) | 10.4 (8.7) | | |
| Severe | 1.3 (1.6) | 1.5 (1.6) | 1.3 (1.1) | | |
| Etiology of FMR, no (%) | | | | 0.950 | 0.032 |
| Non-ischemic | 66.1 (84.8) | 83.1 (84.4) | 99.3 (83.0) | | |
| Ischemic and non-ischemic | 11.9 (15.2) | 15.3 (15.6) | 20.3 (17.0) | | |
| ***Operative*** | | | | | |
| Mechanical valve, no (%) | 58.8 (75.4) | 71.9 (73.1) | 88.5 (74.0) | 0.950 | 0.035 |
| Concomitant procedures, no (%) | | | | | |
| CABG | 10.1 (13.0) | 12.7 (12.9) | 18.6 (15.6) | 0.861 | 0.051 |
| Tricuspid valve repair | 1.3 (1.6) | 19.4 (19.7) | 23.5 (19.7) | 0.002 | 0.409 |
| Other procedures | 7.5 (9.6) | 9.0 (9.1) | 20.1 (16.8) | 0.410 | 0.154 |
| CPB duration (min), median [Q1, Q3] | 97.7 [78.5, 126.6] | 133.0 [119.2, 175.0] | 136.0 [119.0, 158.2] | < 0.001 | 0.630 |
| Cross-clamp time(min), median [Q1, Q3] | 70.1 [55.4, 95.3] | 102.0 [88.7, 127.0] | 97.0 [82.5, 121.6] | < 0.001 | 0.375 |
| ***Early postoperative*** | | | | | |
| Usage of ACEI/ARB, no (%) | 10.7 (13.7) | 14.3 (14.5) | 16.0 (13.3) | 0.973 | 0.023 |
| Operative death, no (%) | 0 | 0.6 (0.6) | 7.6 (6.3) | 0.034 | 0.265 |
| Reoperation for bleeding, no (%) | 0 | 1.1 (1.1) | 11.8 (9.8) | 0.026 | 0.336 |
| New-onset stroke, no (%) | 0 | 0 | 1.0 (0.8) | 0.444 | 0.085 |
| New-onset AF, no (%) | 1.3 (1.7) | 6.6 (6.7) | 8.3 (6.9) | 0.232 | 0.173 |
| Acute kidney injury, no (%) | 6.7 (8.6) | 6.1 (6.2) | 10.6 (8.9) | 0.814 | 0.068 |
| ΔEF (%), median [Q1, Q3] | -5.0 [-11.0, 0.0] | -5.0 [-8.9, 0.6] | -4.0 [-11.0, 2.5] | 0.752 | 0.062 |
| ΔLVEDD (mm), median [Q1, Q3] | -10.0 [-15.0, -7.0] | -11.0 [-15.0, -7.0] | -11.0 [-15.0, -8.0] | 0.423 | 0.080 |
| ΔLAD (mm), median [Q1, Q3] | -9.0 [-12.0, -5.2] | -7.2 [-12.0, -3.0] | -4.0 [-8.0, -1.0] | < 0.001 | 0.483 |
| Tricuspid regurgitation, no (%) | | | | 0.594 | 0.232 |
| No | 36.8 (47.1) | 51.4 (52.2) | 66.0 (55.2) | | |
| Trivial/mild | 41.2 (52.9) | 44.0 (44.7) | 49.4 (41.3) | | |
| Moderate | 0 | 3.1 (3.2) | 4.2 (3.5) | | |
| FMR, no (%) | | | | < 0.001 | 0.826 |
| No | 49.1 (62.9) | 51.5 (52.3) | 117.4 (98.2) | | |
| Trivial/mild | 28.2 (36.2) | 44.9 (45.6) | 2.2 (1.8) | | |
| Moderate | 0.7 (0.9) | 2.1 (2.1) | 0 | | |

Δ Change of echocardiographic characteristics compared to the baseline.

ACEI/ARB, angiotensin converting enzyme inhibitors / angiotensin-receptor blockers; AVR, aortic valve replacement; CABG, coronary artery bypass grafting; CPB, cardiopulmonary bypass; EF, ejection fraction; LAD, left atrial diameter; LVEDD, left ventricular end-diastolic diameter; MVr, mitral valve repair; MVR, mitral valve replacement; NYHA, New York Heart Association; SD, standard deviation; SMD, standardized mean difference.

**Table S4.** Baseline, operative and postoperative characteristics of the subgroup of aortic stenosis in the IPTW analysis.

| Variables | AVR  (n = 78.03) | AVR+MVr  (n = 39.11) | AVR+MVR  (n = 46.57) | P-value | SMD |
| --- | --- | --- | --- | --- | --- |
| ***Preoperative*** | | | | | |
| Age (years), mean ± SD | 59.8 ± 9.9 | 62.0 ± 9.1 | 62.1 ± 9.4 | 0.615 | 0.165 |
| Female sex, no (%) | 44.2 (56.7) | 18.2 (46.4) | 17.0 (36.4) | 0.451 | 0.275 |
| Body mass index (kg/m^2^), median [Q1, Q3] | 21.6 [20.5, 24.2] | 21.8 [20.4, 25.6] | 22.2 [21.0, 23.9] | 0.633 | 0.113 |
| Body surface area (m^2^), median [Q1, Q3] | 1.6 [1.5, 1.8] | 1.7 [1.6, 1.8] | 1.7 [1.6, 1.8] | 0.391 | 0.344 |
| Atrial fibrillation, no (%) | 25.2 (32.3) | 9.5 (24.2) | 5.3 (11.5) | 0.462 | 0.346 |
| NYHA class III or IV, no (%) | 49.7 (63.7) | 25.2 (64.4) | 23.7 (50.8) | 0.606 | 0.185 |
| Hypertension, no (%) | 37.3 (47.8) | 10.4 (26.5) | 15.4 (33.2) | 0.386 | 0.301 |
| Dyslipidemia, no (%) | 13.6 (17.5) | 6.6 (16.8) | 13.5 (29.0) | 0.411 | 0.197 |
| Coronary artery disease, no (%) | 10.6 (13.6) | 5.8 (14.9) | 8.2 (17.5) | 0.903 | 0.073 |
| Diabetes mellitus, no (%) | 6.4 (8.2) | 2.0 (5.2) | 6.1 (13.1) | 0.526 | 0.185 |
| Renal failure, no (%) | 1.5 (1.9) | 1.0 (2.6) | 0 | 0.468 | 0.157 |
| EF (%), median [Q1, Q3] | 57.3 [50.0, 60.0] | 54.3 [40.0, 59.7] | 56.0 [46.2, 60.0] | 0.464 | 0.278 |
| LVEDD (mm), median [Q1, Q3] | 60.2 [52.7, 67.0] | 59.0 [48.0, 65.0] | 61.0 [54.0, 64.0] | 0.707 | 0.202 |
| LAD (mm), median [Q1, Q3] | 44.2 [40.0, 45.0] | 40.9 [40.0, 50.0] | 44.5 [41.0, 48.0] | 0.766 | 0.203 |
| Tricuspid regurgitation, no (%) | | | | 0.825 | 0.310 |
| No | 18.7 (23.9) | 9.6 (24.7) | 17.6 (37.8) | | |
| Trivial | 11.0 (14.1) | 6.6 (16.8) | 7.3 (15.6) | | |
| Mild | 41.1 (52.7) | 17.4 (44.5) | 16.5 (35.5) | | |
| Moderate | 6.8 (8.7) | 5.5 (14.1) | 4.9 (10.5) | | |
| Severe | 0.4 (0.6) | 0 | 0.3 (0.7) | | |
| Etiology of FMR, no (%) | | | | 0.903 | 0.073 |
| Non-ischemic | 67.4 (86.4) | 33.3 (85.1) | 38.4 (82.5) | | |
| Ischemic and non-ischemic | 10.6 (13.6) | 5.8 (14.9) | 8.2 (17.5) | | |
| ***Operative*** | | | | | |
| Mechanical valve, no (%) | 57.9 (74.2) | 20.2 (51.6) | 28.7 (61.6) | 0.271 | 0.319 |
| Concomitant procedures, no (%) | | | | | |
| CABG | 10.6 (13.6) | 5.3 (13.5) | 7.4 (16.0) | 0.957 | 0.046 |
| Tricuspid valve repair | 27.7 (35.5) | 10.8 (27.6) | 9.8 (20.9) | 0.604 | 0.218 |
| Other procedures | 1.5 (1.9) | 1.0 (2.6) | 1.9 (4.0) | 0.722 | 0.083 |
| CPB duration (min), median [Q1, Q3] | 131.7 [95.8, 243.5] | 166.0 [125.8, 178.9] | 155.1 [129.9, 197.5] | 0.826 | 0.121 |
| Cross-clamp time (min), median [Q1, Q3] | 97.0 [66.8, 152.3] | 119.4 [101.0, 148.5] | 119.05 [98.1, 144.8] | 0.725 | 0.333 |
| ***Early postoperative*** | | | | | |
| Usage of ACEI/ARB, no (%) | 7.0 (9.0) | 0.7 (1.8) | 5.6 (12.1) | 0.327 | 0.279 |
| Operative death, no (%) | 0 | 0 | 1.6 (3.5) | 0.408 | 0.179 |
| Reoperation for bleeding, no (%) | 0 | 0 | 2.6 (5.6) | 0.272 | 0.229 |
| New-onset AF, no (%) | 3.1 (4.0) | 0.3 (0.7) | 8.4 (18.1) | 0.004 | 0.436 |
| Acute kidney injury, no (%) | 8.3 (10.6) | 8.4 (21.5) | 9.1 (19.5) | 0.615 | 0.201 |
| ΔEF (%), median [Q1, Q3] | -3.5 [-12.5, 4.8] | 2.0 [-3.0, 6.0] | -1.0 [-5.7, 5.7] | 0.689 | 0.272 |
| ΔLVEDD (mm), median [Q1, Q3] | -11.0 [-16.28, -6.0] | -6.0 [-13.00, -2.0] | -11.0 [-14.0, -4.1] | 0.340 | 0.297 |
| ΔLAD (mm), median [Q1, Q3] | -7.4 [-9.0, -5.0] | -4.0 [-13.0, 0.0] | -5.0 [-9.0, -0.1] | 0.343 | 0.332 |
| Tricuspid regurgitation, no (%) | | | | 0.305 | 0.423 |
| No | 52.4 (67.2) | 17.7 (45.2) | 27.3 (58.6) | | |
| Trivial/mild | 22.2 (28.5) | 21.4 (54.8) | 19.3 (41.4) | | |
| Moderate | 3.4 (4.3) | 0 | 0 | | |
| FMR, no (%) | | | | 0.075 | 0.573 |
| No | 57.8 (74.1) | 34.3 (87.7) | 46.6 (100.0) | | |
| Trivial/mild | 18.5 (23.7) | 4.5 (11.4) | 0 | | |
| Moderate | 1.7 (2.2) | 0.3 (0.9) | 0 | | |

Δ Change of echocardiographic characteristics compared to the baseline.

ACEI/ARB, angiotensin converting enzyme inhibitors / angiotensin-receptor blockers; AVR, aortic valve replacement; CABG, coronary artery bypass grafting; CPB, cardiopulmonary bypass; EF, ejection fraction; LAD, left atrial diameter; LVEDD, left ventricular end-diastolic diameter; MVr, mitral valve repair; MVR, mitral valve replacement; NYHA, New York Heart Association; SD, standard deviation; SMD, standardized mean difference.
